# The power of one – in memoriam of Paul E. Farmer

**DOI:** 10.1002/jia2.25903

**Published:** 2022-04-06

**Authors:** Rajesh Gupta, Serena P. Koenig

**Affiliations:** ^1^ Harvard Medical School Brigham and Women's Hospital USA

Paul Farmer was one of the fiercest social justice advocates for the poor and underserved in modern medicine. As a physician, anthropologist and activist, he witnessed the intricate interplay of social and economic disparities on health outcomes [1]. With an unrelenting drive that spanned decades, he questioned the assumption that the world could not provide the same level of high‐quality healthcare for the poor that we provide to the rich, and he argued that we replace this “socialization for scarcity…for black and brown people” with a vision of equity [2]. With collaborators, he created his vision of a “preferential option for the poor” [3] through a series of successful interventions: providing antiretroviral therapy (ART) for persons living with HIV in Haiti; treating drug‐resistant tuberculosis (TB) in Peru and Russia; transforming the healthcare system in Rwanda; establishing Ebola treatment centres in West Africa; and, ultimately, translating decades of experience implementing community‐based healthcare approaches into improving the COVID‐19 response in the United States. Paul's integration of socio‐medical interventions fundamentally altered the approaches and principles for treating infectious diseases globally [4].

Paul's efforts were driven by an outrage against the global health community's complacency around providing healthcare services to the poor.  As he saw a broad acceleration of innovative ideas in healthcare and an unprecedented increase in wealth in many countries, he repeatedly questioned why global health aspirations remained stagnant and why policy makers devalued the lives of the poor by claiming resources were scarce or that it was not feasible, realistic or prudent to provide the same high‐quality care to all.  As an example, he told a story about his patient who was a prisoner in Siberia living with HIV. This patient had deliberately infected himself with drug‐resistant TB just so he could qualify for a paltry daily meal. A policy maker recommended withholding treatment for both diseases based on cost‐effectiveness calculations, when it was entirely possible to treat that patient. Paul, rejecting the notion that the lives of some were subjected to economic decisions while the lives of others were not, had collected a mental library of these types of policy decisions to counteract – one by one.


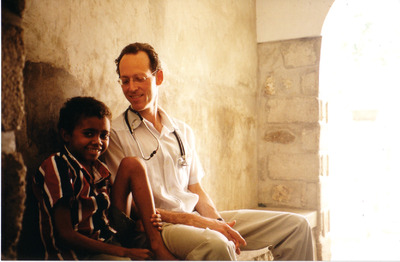


**Dr. Paul Farmer with a patient in Cange, Haiti, 2000 (photo by Moupali Das for Partners In Health)**.

Paul's goals were always clear – to “counter failures of imagination” and to overcome the barriers to health equity with the “5 Ss”: staff, stuff, space, systems and social support [5]. One powerful illustration of this approach is the HIV Equity Initiative, which was started by Paul and others at Partners In Health (PIH) in Haiti in the late 1990s. At a time when millions of people were dying of HIV in Africa, but many experts professed that providing HIV treatment in low‐income settings was too complicated and expensive, Paul obtained the necessary medications and implemented the same strategy that had been effective in TB – directly observed treatment and social support with community health workers (known as *accompagnateurs)*. Within a short time, cachectic patients with advanced AIDS experienced the “Lazarus effect” and returned to vigorous health. Paul, through PIH and in collaboration with others, scaled up this experience in multiple countries, including Rwanda and Lesotho; these programs provided justification for the broader scale‐up of ART in all settings globally [6]. Always bringing in his wry sense of humour, he once confided “Can you believe we actually had to do a study to convince people that if you are sick and you get effective treatment, then you get better ‐ even if you are poor?”

Balancing his anger at the injustices of the world, though, was his “unconditional kindness” and his wise, generous and influential mentorship [7]. Paul made everyone feel important, and once you interacted with him, you had a friend for life who picked up right where your last conversation left off – whether you spoke with him regularly or once every few years [8, 9]. We each met Paul roughly 25 years ago, at early points in our careers. He gave us the moral imperative to become the social‐justice version of whatever we wanted to be in life. He pushed us to do our best, because the poor deserved nothing less and lives were at stake.  There are countless others who were mentored by Paul, and who were profoundly affected in similar ways. Many have commented that the lessons they learned from him made them better people, and they try to pass these lessons on to those they mentor. Several of Paul's former mentees and colleagues described a persistent sense of security in knowing that he was there, leading the fight for global health equity, and are feeling unmoored since his death. His moral clarity, humility, persistence in approaching the work of service, optimism and faith in humanity – these are things we now carry with us. His influence continues to shape and define our work, whether we are seeing patients, developing therapeutics and vaccines, or performing clinical research.

Through his prolific writings and speeches, Paul inspired thousands more across a diverse range of backgrounds and disciplines. Paul made it clear that everyone has an essential role in the fight for social justice, under an umbrella of solidarity, regardless of one's discipline, area of expertise or occupation. He said: “I am [actually] not enthralled with any particular discipline of study…[just] with what one might call corrective medicine and public health….if what you want to do is serve and repair the wounds of this world, then we share that much.” Arguably, this is how a movement is built.

What do we do when someone with impact and influence so deep and vast is no longer with us? How do we advance his vision? Paul's answer to this question would likely be something simple and obvious: aggressively move forward to achieve global health equity. Fortunately, through his writings, teachings, individual conversations and practice, Paul has left a roadmap for the future of this movement [10].

Clearly, the fight for global health equity has only just begun as the “socialization of scarcity” remains dominant. The ongoing COVID‐19 pandemic exposes continuing structural violence against the poor: inequitable policies by those in power to create the highest standard of prevention and treatment for the wealthiest and least vulnerable, and the lowest standard for the poorest and most vulnerable.  As we honour and carry forth Paul's work, it is up to all of us to refuse to be complicit and complacent in accepting such inequities. It is up to all of us to continue relentlessly fighting for the poor and underserved.

## COMPETING INTERESTS

RG is an employee of and may own shares in Vir Biotechnology, Inc., which is developing vaccines and therapies for global infectious diseases. SPK receives grant funding from ViiV and Gilead to her institution, and is an ad‐hoc consultant for Analysis Group. No companies were involved in the drafting or review of the manuscript. All ideas and opinions expressed in the manuscript are strictly of the authors.

## AUTHORS’ CONTRIBUTIONS

RG and SPK developed the concept and wrote all drafts of the manuscript.

## Data Availability

There were no data used in this manuscript.
